# Enablers to Exercise Participation in Progressive Supranuclear Palsy: Health Professional Perspectives

**DOI:** 10.3389/fneur.2020.635341

**Published:** 2021-02-02

**Authors:** Meg E. Morris, Susan C. Slade, Christopher Bruce, Jennifer L. McGinley, Bastiaan R. Bloem

**Affiliations:** ^1^La Trobe Centre for Sport and Exercise Medicine Research, School of Allied Health, Human Services and Sport, La Trobe University, Bundoora, VIC, Australia; ^2^Healthscope Academic Research Collaborative in Health (ARCH), Victorian Rehabilitation Centre, Glen Waverley, VIC, Australia; ^3^Discipline of Occupational Therapy, School of Allied Health, Human Services and Sport, Science, Health and Engineering College, La Trobe University, Bundoora, VIC, Australia; ^4^Physiotherapy, The University of Melbourne, Parkville, VIC, Australia; ^5^Radboud University Medical Centre, Nijmegen, Netherlands; ^6^Donders Institute for Brain, Cognition and Behaviour, Nijmegen, Netherlands; ^7^Department of Neurology, Centre of Expertise for Parkinson and Movement Disorders, Nijmegen, Netherlands

**Keywords:** progressive supranuclear palsy, exercise, nursing, allied health, qualitative research

## Abstract

**Background:** People living with progressive supranuclear palsy (PSP) can experience considerable difficulties with movement, walking, balance, and oculomotor control. The role of exercises and physical activities in mitigating the motor and non-motor symptoms of PSP remains uncertain.

**Aims:** The aim of this study was to identify the perspectives and beliefs of health professionals about the benefits, enablers, and barriers to participation in exercise and physical activity across the course of disease progression of PSP.

**Methods:** Qualitative methods, within a phenomenological framework, were used to obtain nursing and allied health professional perspectives and recommendations. Focus group and in-depth interview questions were derived from a systematic review on exercise for PSP. Expert opinions also guided the interviews, which were audio-recorded, transcribed verbatim, and de-identified. Two researchers independently conducted a thematic analysis.

**Results:** Nineteen health professionals participated from the disciplines of nursing, physiotherapy, occupational therapy, and speech pathology. Four main themes emerged: (i) exercise and physical activities are important for living well with PSP; (ii) provision of information about the benefits of exercise and physical activities facilitates uptake; (iii) interdisciplinary teams work together to improve outcomes; and (iv) care partners can assist with the implementation of exercise and physical activities.

**Conclusion:** Health professionals advocated physical therapies for people living with PSP. The expectation is that structured exercises and physical activities can help to optimize health and well-being, enabling people to continue to participate in social roles. The actual merits of such interventions must now be tested in large-scale controlled clinical trials.

## Introduction

Progressive supranuclear palsy (PSP) is one of the most debilitating forms of atypical parkinsonism (1, 2). Affecting around a million people worldwide (3), PSP can be associated with a rapid deterioration in balance, gait, movement, and oculomotor control (4). Non-motor symptoms, such as fatigue, anxiety, and depression, are also common (5, 6). Falls are a prominent feature of PSP and become more frequent as the disease progresses (7). It has been estimated that over 80% of people living with PSP experience falls (7, 8). Currently, there is no cure for PSP, and even symptomatic support can be challenging.

Symptomatic treatment is based on alleviating specific symptoms or signs and aims to optimize health, well-being, and quality of life (9). Treatment also aims to reduce negative sequelae, such as falls and social isolation. Nurses and allied health professionals play key roles in implementing evidence-based interventions, alongside medical management, for people living with PSP (10). This includes exercise, physical activities, and movement rehabilitation, which are cornerstones for managing parkinsonism and related disorders (11).

Structured exercises can improve strength, mobility, and balance in people living with PSP (9). Physical therapy (11) and physical activities, such as walking (12, 13), music-cued movement (14), dancing (15), and cycling (16), can also improve fitness, community ambulation, and social participation in parkinsonism. Physiotherapy, occupational therapy, and speech pathology also play a role in the comprehensive care of people with progressive neurological conditions, such as PSP (9).

One of the challenges for nurses and allied health professionals is to know how best to facilitate exercise, physical activities, and movement rehabilitation in people living with PSP. In a recent study on barriers and facilitators to exercise, people with PSP and their care partners reported that long delays in receiving a definitive diagnosis of PSP had a negative impact on exercise participation. Access to allied health and nursing professionals with expertise in PSP was also a barrier. Because each person with PSP has a unique array of symptoms and co-morbidities, a need was expressed for access to specialist care. The care provided also needs to consider the stage of disease progression (9, 10).

What are not known are the views of nurses and allied health professionals about the factors that facilitate and enable participation in structured exercises, physical activities, and motor skill training across the course of disease progression in PSP. This study aims to address that need through in-depth interviews with nurses, physiotherapists, occupational therapists, and speech pathologists.

## Methods

Ethical approval was granted by the La Trobe University human ethics committee (HEC18333). All participants received a Participant Information Sheet and provided written informed consent prior to data collection. Each participant was assigned a code number and advised that all information was confidential and anonymous. Hard copy consent forms, demographic data, and participant master list were stored in a locked filing cabinet at the university. The audio-recordings, de-identified transcriptions were stored on a secure university research drive. Data collection consisted of a mixture of focus groups and in-depth individual interviews according to workplace and participant availability. The Consolidated Criteria for Reporting Qualitative studies (COREQ) checklist was used to inform study design and reporting (17).

### Theoretical Framework

A phenomenological theoretical framework was selected to explore the lived experience of the participants (18, 19). This approach allowed a deep understanding and exploration of participant beliefs and experiences in providing guidance about exercise participation for people living with PSP.

### Research Team

The study team included clinical and research experts in parkinsonism and movement disorders (MM, JM, BB). It also included allied health professionals with qualitative research skills (SS, CB) and exercise prescription expertise (SS, MM, JM). Members of the research team were not known to the participants.

### Participant Eligibility and Recruitment

Registered nursing and allied health professionals were recruited *via* purposive sampling in Melbourne, Australia until data saturation was reached. There was representation of the health professionals most likely to advise people living with PSP about exercise and physical activity. These included nurses, occupational therapists, physiotherapists, and speech pathologists. Movement disorders clinics, neurological rehabilitation clinics, private practices, the not-for profit sector, and the Parkinson's association were approached by email and/or telephone and provided with information about the research.

### Data Collection

An experienced facilitator (SS), who was not known to the participants, conducted the audio-recorded focus groups and individual interviews. A set of interview questions was informed by a systematic review (9), exercise audits (20), expert opinions from the research team, and consultation with the Parkinson's association representatives (Table 1). Demographic data were collected about age, qualifications, and experience with movement disorders and PSP. Field notes were made immediately after each focus group and interview to provide a record of observations and impressions. Data collection took place from October 2018 until April 2020.

**Table 1:** Interview question guide.

1. What has been your experience with the diagnosis of PSP?
2. What information do you give about management options?
•Exercise
•Physical activity
•Information access, assistance, sources
•Assistive devices for activities of daily living
3. What do you believe/think about exercise, physical activities, and movement strategies for people with PSP?
4. What do you think would help people with PSP to participate in exercise?
5. Do you know where or how to access information?
6. How do you access the research evidence about PSP and exercise?
7. Do you use Parkinson's disease literature and evidence to inform your management of PSP?


### Data Analysis

Two researchers (SS and CB) independently conducted inductive, step-by-step thematic analysis of the de-identified transcripts (using data immersion, coding, and categorization) (18, 19, 21). They met regularly by telephone and videoconferencing to discuss and document coding and theme development until consensus was reached. Two audio-recorded meetings were convened with CB, MM, and SS to reach final consensus of themes and sub-themes. A range of participant quotations with code number and transcript/line number were selected to support the themes.

### Control of Bias

Control of risk of bias was enhanced by defined eligibility criteria; participant recruitment that provided a range of experiences to answer the research question, reproducible data collection, and analysis steps; audio-recording and verbatim transcription; independent data analysis by two researchers and team consensus; and findings supported by participant data (22, 23).

## Results

There were 19 participants, and the overall saturation of ideas and findings was achieved in the total pool of participants. Two focus groups were conducted with a total of seven movement disorders physiotherapists. One focus group was also conducted with two speech pathologists, and an additional focus group was conducted with two occupational therapists. All of the clinicians worked in movement disorders in the public sector. In-depth individual interviews were additionally conducted with a total of eight health professionals working in movement disorders in the public, private, and not-for profit sectors (four nurses, three physiotherapists, and one speech pathologist). The interviews and focus groups were facilitated by SS in the participant workplaces. They were audio-recorded and transcribed verbatim. The focus groups had a mean duration of 65.8 min and standard deviation of 15.9 min. The individual in-depth interviews had a mean duration of 55.5 min and standard deviation of 10.7 min. Participant demographic data are summarized in Table 2. We included more physiotherapists because they typically provide exercise and activity instruction to people living with PSP and Parkinson's disease.

**Table 2:** Participant characteristics.

**Code**	**Profession**	**Qualification**	**Years of experience**	**Healthcare sector**	**Full time or part time**	**Expertise**	**Interview time (min)**
1	PT	Bachelor of Physiotherapy	30	Private	P/T	Neurological rehabilitation, movement disorders	FG 1: 83
2	PT	Bachelor of Physiotherapy, Master of Neurological Rehabilitation	22	PrivatePublic	P/T	Neurological rehabilitation, movement disorders	FG 1: 83
3	PT	Bachelor of Physiotherapy	8	Private	F/T	Neurological rehabilitation	FG 1: 83
4	PT	Bachelor of Physiotherapy with honors	9	Public		Movement disorders	59
5	PT	Bachelor of Science (Physiotherapy)	4	Public	F/T	Neurological, movement disorders	FG 2: 73
6	PT	Bachelor of Physiotherapy	11	PrivatePublic	P/T	Neurological rehabilitation	FG 2: 73
7	PT	Bachelor of Physiotherapy	4	PrivatePublic	P/T	Neurological rehabilitation, movement disorders	FG 2: 73
8	PT	Bachelor of Health Sciences, Master of Physiotherapy	6	Public	F/T	General inpatient, movement disorders	FG 2: 73
9	SP	Bachelor of Applied Science (Speech Pathology)	30	Non-government organization	P/T	Movement disorders	74
10	N	Diploma in Applied Science (Nursing), Bachelor of Science in Nursing, Master of Science in Nursing	30	Non-government organization	F/T	Movement disorders	52
11	PT	Bachelor of Physiotherapy	15	Private	F/T	Neurological rehabilitation	57
12	PT	Bachelor of Applied Science (Physiotherapy), Graduate Diploma in Neurology, PhD	39	PublicPrivate	P/T	Neurological rehabilitation, academic, research	61
13	N	Bachelor of Science in Nursing, Graduate Certificate in Community Health	39	Public and non-government organization	P/T	Movement disorders 15 years	38
14	N	Diploma in Nursing, Bachelor of Science (Clinical Nursing), Nurse Prescriber	20	PublicPrivate	P/T	General nursing, movement disorders nurse (5 years)	57
15	N	Bachelor of Science in Nursing, Graduate Diploma in Nursing (CCU)	28	Public	F/T	General nursing, movement disorders nurse (12 years)	46
16	SP	Bachelor of Medical Sciences (Speech Pathology) with honors	14	Public	P/T	Neurological rehabilitation, movement disorders	FG 3: 61
17	SP	Bachelor of Applied Science (Speech Pathology)	40	Public	P/T	Neurological rehabilitation, voice disorders	FG 3: 61
18	OT	Bachelor of Applied Science (Occupational Therapy)	17	Public	P/T	Neurological rehabilitation, movement disorders	FG 4: 46
19	OT	Bachelor of Applied Science (Occupational Therapy)	21	Public	P/T	Neurological rehabilitation, movement disorders	FG 4: 46

*N, nurse; OT, occupational therapist; PT, physiotherapist; SP, speech pathologist; F/T, full time; P/T, part time; FG, focus group*.

Data were stored on a secure university research drive that could be accessed only by the lead researcher and the facilitator. There were perspectives and experiences common to all of the participants, with no dissonant cases. Saturation was reached after 19 participants who all volunteered their ideas and beliefs. Member checking of the transcripts was not conducted because of the risk of recall bias.

The overarching theme to emerge from this study was that nursing and allied health professionals believed that exercise, physical activities, movement rehabilitation, and functional activities are important components of therapy to enable people with PSP to live well. Nevertheless, there was uncertainty about exactly how these should be delivered and how they should be tailored to individual needs and the stage of disease progression.

Four main themes were identified: (i) exercise and physical activities are important for living well with PSP; (ii) provision of information about the benefits of exercise and physical activities facilitates uptake; (iii) interdisciplinary teams work together to improve outcomes; and (iv) care partners can assist with the implementation of exercise and physical activities. The themes and accompanying sub-themes are presented in the following text and supported by quotations linked to the participants by code number and transcription line numbers.

### Theme 1: Exercise and Physical Activities Are Important

Exercise and physical activity engagement were viewed as being helpful for health and wellbeing, especially in the early stages of disease progression, and when adjusted to individual needs and preferences.

#### Health Professionals Recommend Individualized Programs

The participants reported that exercise and physical activity are best tailored to each individual with PSP, their physical capacity, preferences for exercise, and prior exercise participation (Box 1). They also advised that there are many different modes, models, and approaches to exercise, physical activity, and movement rehabilitation for people living with PSP.

BOX 1 “*The model of care we used was always to ask the person their priority.” P1, line 1,133*

“*It helps if they've had an experience with exercise then they know what's involved.” P2, line 489*

“*Some people are gym people, some people are not gym people. Some people like exercising in groups, some people don't, so we'll get their thoughts on what sort of exercise they would like to do.” P4, line 117–120*

“*It's all patient dependant... if it was someone who came in that has been to the gym all their life, and their want is to have exercise is better to do with using dumbbells or some weights, then they will get those exercises to do, because that's what drives them.” P7, line 157*

“*I might take photos of them doing the exercise... laminate it and send it out to the patient. And they love that. Because they're, ‘Right that’s me. I can do that'.” P12, line 361*

“*Music engagement, interaction with people, something that's fun to do, it doesn't seem like boring lifting weights or anything, but you still get muscles working.” P15, line 642*

“*I'm not sure that I've got a sense with people with this particular disorder of what might be best. I tend to think maybe they're going to have to have individualized things.” P11, line 665*

“*We started the groups... the cost of the class is significantly less. So the idea was to be able to keep people coming along and exercising in a group environment.” P12, line 218*

“*It's finding something that they enjoy that they might actually do*.” *P16, line 811*

#### Early Therapy Is Advisable in PSP

The clinicians also believed that exercise early in the time course of PSP is important for optimizing strength and motor function. They advised that it would be easier to introduce before potential complications of cognitive impairment (Box 2).

BOX 2 “*The good thing about early diagnosis and treatment is that there's not, usually there's not as many things going on, so you can focus on starting to implement exercise as a starting block. If you can start early enough, then they can focus on that because they don't have many other things to focus on.” P5, line 967–970*

“*I think the other issue with early vs. late referrals is because there are so many more barriers later, if you are getting referred someone who has those barriers and hasn't exercised in the 3 years of their diagnosis, it's very difficult to make that change.” P3, line 796–798*

“*It would be great to get them in early when they can really participate quite actively in the group... building up strength before losing it, or getting as strong as possible and moving more earlier, rather than waiting for the decline to happen, to then try and maintain.” P12, line 58*

“*I think coming back to that early prescription for exercise and activity for people is a great idea and that's something that I think that could be a game changer for a lot of people.” P15, line 581*

#### Exercise and Physical Activity Can Be Empowering

The clinicians advised that maintaining exercise and fitness could be empowering and beneficial to health, including over the long term (Box 3).

BOX 3 “*It's (exercise) something that is a tangible thing they can do. I think it really does matter that you give them a sense of empowerment that they are doing what they can do.” P3, line 307*

“*I think strength training is important for anyone. You want to maintain someone's function and mobility as much as possible within this condition. For function–bed transfers, chair transfers, walking–I feel there's a place for strength training within an exercise program.” P4, line 767*

“*She certainly has some other issues with arthritis and so forth. That had an impact on the rate of deterioration... some of that disability wasn't from PSP. It was due to weakness.” P11, line 321*

“*I think there's the physical aspect (of exercise), the physical benefits, the cardiac benefits—just being mobile is so much better for all your body systems... for the psychological, the sense of being able to do some physical activity... Sense of achievement, just the fact that they can achieve something themselves.” P13, line 431*

“*The stronger they are, the better their muscles are, and point out that when people—when we're younger and we lose our balance, we don't always fall because we can correct our balance because we have good muscles.” P15, line 138*

#### Health Professionals Recommended Incorporation of Exercise Into Daily Life

Participants discussed the importance of finding regular times for structured exercise, as well as finding individual motivators for exercise. It was recommended that people incorporate exercise into daily activities or a home program, to become a routine activity (Box 4).

BOX 4 “*You try and drum into anyone in our program that those people that exercise regularly and consistently do have better outcomes functionally than those that don't. We try and link their goals at home to exercise.” P4, line 222*

“*It's the advice on finding exercise that works for you that is less of chore, that you can do every day.” P12, line 307*

“*The idea was to try and make things (exercises) as normal as can be in such a situation but not to sort of turn it into another therapy room.” P11, line 228*

“*Ensure... does 20 minutes of bike every day, and does five minutes of arm exercises every day and then aims for 10,000 steps a day.” P5, line 745*

“*The ones who don't want to exercise, I would encourage them to do those sit to stands every now and again, and those functional movements to encourage them to stay as independent and safe as possible.” P7, line 163*

“*It was football that he played as a younger man, and was passionate about sport. So sport and exercise was his thing. So going to the gym on a frequent basis was part of their regular week.” P9, line 509*

“*Each day or five days a week... you need to spend a bit of time doing exercise each day.” P12, line 307*

### Theme 2: Information About the Benefits of Exercise and Physical Activities Facilitates Uptake

#### Health Professionals Need Access to Knowledge and Resources

All of the health professionals agreed on the importance of access to evidence-based information and resources supporting therapy for PSP. This was sometimes challenging due to the small amount of research on physical therapies for PSP and the absence of clinical guidelines on exercise for PSP (Box 5).

BOX 5 “*I've found that from an exercise and a speech pathology point of view, it's hard to know what to recommend. I've searched for guidelines and not really found anything clear.” P17, line 247*

“*In terms of treatment, they mostly get treated as per the Parkinson's patients. We run Parkinson's groups and we would have PSP patients in the Parkinson's groups.” P7, line 80*

“*I'll definitely use Dr Google first of all, but I will be looking for national organizations and things like that. Cochrane I find very helpful if I just want to get a reasonably quick idea of what's out there and also probably Pub Med.” P11, line 590*

“*I'm probably not that good at accessing all the research... But as far as relaying that–relaying information from–on research papers on the issue of exercise, it's mainly been informal learning.” P13, line 200*

“*From an OT perspective, we tend to look at things from a more practical perspective. I always try and see whether the Parkinson's skills or strategies are transferable to someone with PSP, and either it works or it doesn't.” P18, line 320*

“*There just hasn't been a lot out there PSP-specific to get to. It's great that it's available but when you need that specific information, it can be hard to find.” P4, line 698*

#### Timing and Methods of Information Delivery Are Key

The participants also advised that timing the delivery of exercise information to people living with PSP should take into account the stage of disease progression, individual needs, and suitable amounts of information (Box 6).

BOX 6 “*At the start, it's a very tricky thing for a lot of people to come to terms with, having a diagnosis of PSP compared to Parkinson's, so you don't want to overload them. It's more just taking a graded approach, and identifying where they are in that journey, at what stage, and the most appropriate things that they need at that point.” P4, line 51–55*

“*Sometimes people actually don't want too much information early, and we've got to be so sensitive to where are they in what they want, and what they can manage... there's a lot of inaccurate information out there.” P9, line 234*

“*The manual that we have has got a whole range of—all the different symptoms that can be experienced. Some of us in the health team are more reluctant to send out the whole entire folder. I think the others in the team feel that it's too much information, so it is better to trickle out the information as the time moves on.” P13, line 71*

“*One of the things that we do with our clients here as a priority, is to encourage health literacy, so the minute they come to our service, we're starting to empower them.” P17, line 626*

#### Access to Information Is Variable

The clinicians in this study indicated that access to reliable information through formal research and education channels was sometimes a challenge for health professionals. Regular staff meetings and informal and formal professional networks and special interest groups were helpful for knowledge transfer (Box 7).

BOX 7 “*I do get calls from physios around town, calls or emails saying do you know anyone who knows anything about this condition.” P1, line 146*

“*Through Parkinson's XXXX, we have the physiotherapist group that meets every two months. If I've got questions, I will just email people through that and say, “Do you have any more information about this?” or, “What do you think about this?” Bounce back a lot on the colleagues.” P4, line 678*

“*It has an—that's right, emotional toll, and you do. You come back and you'll debrief with your colleagues within the team. You do have to get it off your chest a little. It's quite emotionally draining.” P4, line 889*

“*We normally have our own physio meeting, between us physios once a week … to talk about anything's to raise, anyone's got any concerns, anyone's got a problem with a patient or needs a bit of brainstorming.” P5, line 228*

“*There's also hospital wide, sometimes a neurologist would run specific in services and things, about different topics, and we're always free to ask them to talk about certain topics if we want to.” P6, line 411*

“*Part of our service is supposed to be educating local therapists as well, as part of our service, we do have a role in what they call secondary consultation, so people can call us about a patient that we don't know about, for advice et cetera.” P6, line 1,058*

“*We're also quite lucky that our gym was quite large, and everyone treated in the same place. We were able to, even watch and hear how people coach patients … you could learn from not just seniors, but just other colleagues and juniors, on how different things are being said and done, and not just technical skills.” P7, line 265*

“*We do them (in-services) monthly, but they go for an hour and a half. So we really try and delve into a topic and change our clinical practice.” P12, line 800*

“*Every client, we would tell them to contact Parkinson's XXXX. I can't think of a client that we wouldn't say that to, unless they'd already told us that they've been there done that. So it's very much our standard practice to alert them to it.” P16, line 214*

### Theme 3: Interdisciplinary Teams Work Together to Improve Outcomes

Nursing and allied health professionals discussed having a range of skills to offer and noted how interdisciplinary communication and teamwork can be beneficial (Box 8).

BOX 8 “*I personally say that amongst our multidisciplinary health team with physiotherapist, speech pathologist, two nurse consultants, we have many years of experience.” P10, line 51*

“*So we'll refer on to OTs and speechies and neuropsychs who are probably the main loop that we have. And depending on the client, we might have team meetings… We were talking earlier about linking up with musculoskeletal colleagues more.” P12, line 184*

“*It's very important to be reviewed by an occupational therapist or a physiotherapist, to not go out and buy anything (assistive devices) until they've been assessed.” P13, line 391*

“*Unless they come to us later and it's very obvious, then we'll talk about the diagnosis and very quickly talk about allied health as the most important aspect of their management.” P36, line 74*

“*Once I've found out what the physio's recommendations are, then I can reinforce them. That's how I see my role.” P17, line 334*

### Theme 4: Care Partners Can Assist With the Implementation of Exercise and Physical Activities

#### Care Partners Have Variable Levels of Knowledge, Stress, Fatigue, and Resilience

All of the participants acknowledged that care partners can be a valuable asset in providing advocacy and re-enforcement of care plans and exercise programs. The clinicians provided resources and explanations to educate care partners and emphasized the benefits of support groups. However, there were variable levels of care partner knowledge and support. Individual characteristics of resilience and being proactive were seen as facilitators to successful care giving for people living with PSP (Box 9).

BOX 9 “*Having a family member there, or a carer there means that that person will be able to implement the recommendations at home. Also, they can be more actively engaged in therapy or intervention that you're offering, so they can be a therapy partner.” P16, line 74*

“*If you've got a really supportive partner that's engaged and helping to kind of overcome some of those barriers that makes a huge difference or family support of some kind.” P3, line 591–592*

“*Our social worker a few times a year does run a carer support group for ten weeks... They feel like they're stretched thin, carers.” P4, line 251*

“*Another factor to account for is their cognition and that environment that might support them best, and whether their carer is trained to really accommodate, not just the new exercises or the exercises we might be asking them to do.” P8, line 199*

“*And the number of calls that we have that will say, “Where can I meet other people with this condition?” Or “As a carer I'm really struggling, where can I go? Where can I go to meet with other people who can support me?” P9, line 170*

“*The major issue we always stumble on is trying to find a practice partner or a someone who is not their main caregiver because the idea I've always worked on is trying to give the caregiver a bit of a break and not always be responsible for things.” P11, line 156*

“*We are pretty big on getting patients to bring someone with them to the appointments, so that they can take all that information away as well.” P12, line 295*

“*A couple of partners of people with PSP were still coming (to support groups) after their partner had died. They were still attending the group because they had formed a connection.” P13, line 357*

“*We have a carers group that meets once a month where they can come and have some time talking and discussing with other people in the same situation as themselves.” P15, line 92*

## Discussion

Nurses and allied health clinicians expressed positive views about the contribution of structured exercises, physical activities, and movement rehabilitation for people living with PSP. Especially in the early stages of the condition, physical therapies were argued to improve health and quality of life. Some professionals advised that high intensity structured exercises might slow the rate of disease progression in people living with PSP, in agreement with studies hinting at the possibility of neural plasticity in Parkinson's disease and related conditions (24, 25). For example, a meta-synthesis of the literature on exercise in Parkinson's disease by Johansson et al. (26) reported that brain function and brain structure can alter in a positive way with intense and sustained exercise. Likewise, Frazzitta et al. (24) showed that high intensity and individually tailored physiotherapy and exercise programs were associated with plastic changes of benefit to health and well-being. Liu (25) reported that physical exercise can modulate the pathophysiological mechanisms of neurodegeneration in Parkinson's disease, although it remains unclear how this affects disease progression. Moreover, a recent home-based exercise study showed that aerobic exercise (cycling on the stationary trainer for 30–45 min, three times per week) was associated with a stabilization of off-state motor signs in people with Parkinson's disease, compared with a control group that performed stretching exercises (16). Further work is needed to determine whether such clinical effects result from symptomatic improvements or reflect adaptive plasticity that could translate into a disease modifying effect.

Health professionals in the current investigation cited barriers and facilitators to exercise participation in people living with PSP. A barrier to the uptake of physical therapies was difficulty in accessing evidence-based resources. This was particularly challenging for PSP due to the paucity of research and the absence of clinical guidelines for this progressive neurological condition. In the absence for this evidence-based resource, nurses, and allied health professionals often turn to the European Clinical Guidelines for Parkinson's Disease to help inform therapy content, dosage, and mode of delivery (9, 10, 20, 27). There is an urgent need for global clinical guidelines for exercise and physical activity in PSP. This needs to address the relative contributions of gait training (13), falls education (12, 28), balance exercises (29), progressive resistance strength training (12), strategies to improve motor skills (30), and structured physical activities, such as dancing (15), aquatic therapy (31), cycling (16), boxing (27), virtual reality exercises (32), and online home exercises (33, 34).

Facilitators to exercise in PSP included an early diagnosis, information on individual goals, access to structured exercise, and professional advice from therapists skilled in the management of PSP and related conditions. Furthermore, the effectiveness of treatment was enhanced through regular staff meetings and informal and formal professional networks and special interest groups. In other disciplines, teaching for example, professional learning networks have been reported to enhance a sense of worker autonomy, as well as increasing motivation, by offering access to support and information both locally and on a global scale (35).

There were several limitations to this study. The health professional participants who were recruited had experience with PSP and were actively involved in PSP care. This situation is probably different among generically trained therapists who have less expertise with this complex condition. Although we interviewed nurses, physiotherapists, occupational therapists, and speech pathologists, we did not obtain the views of movement disorders neurologists and some other allied health professionals, such as psychologists, dieticians, social workers, and exercise scientists. This would be very helpful in developing comprehensive inter-professional guidelines for the management of PSP. With regard to response bias, participants may have responded in a way that may have overemphasized their views, given the aim of the study was to investigate participation in structured exercises and physical activities. Additionally, more needs to be done to better understand the content of exercise programs specifically for PSP, optimal delivery modes, and how programs should be tailored depending on the disease stage.

To conclude, exercise and physical therapies appear beneficial for people living with PSP and have the potential to improve health and well-being in this debilitating condition. Co-producing evidence with people living with PSP and their care partners on the effects of physical therapies is a high priority for implementation research.

## Data Availability Statement

The raw data supporting the conclusions of this article will be made available by the authors, without undue reservation.

## Ethics Statement

The studies involving human participants were reviewed and approved by La Trobe University Human Ethics Committee (Project: HEC18333). The patients/participants provided their written informed consent to participate in this study.

## Author Contributions

MM and SS conceived the idea for the study. MM, SS, CB, and BB were responsible for the study design, study implementation, writing, manuscript revisions, and read and approved the final manuscript. MM was guarantees that the authorship statement is correct. All authors contributed to the article and approved the submitted version.

## Conflict of Interest

The authors declare that the research was conducted in the absence of any commercial or financial relationships that could be construed as a potential conflict of interest.

## References

[1] Stamelou M Hoeglinger GU. Atypical Parkinsonism: an update. Curr Opin Neurol. (2013) 26:401–5. 10.1097/WCO.0b013e3283632da6 23812308 PMC4196800

[2] Höglinger GU Respondek G Stamelou M Kurz C Josephs KA Lang AE Clinical diagnosis of progressive supranuclear palsy: the Movement Disorder Society Criteria. Mov Disord. (2017) 32:853–64. 10.1002/mds.26987 28467028 PMC5516529

[3] Stamelou M Giagkou N Hoglinger GU. One decade ago, one decade ahead in Progressive Supranuclear Palsy. Mov Disord. (2019) 34:1284–90. 10.1002/mds.27788 31283855

[4] Rezvanian S Litvan I Standaert D Jankovic J Reich SG Hall D. Understanding the relationship between freezing of gait and other progressive supranuclear palsy features. Parkinsonism Relat Disord. (2020) 78:56–60. 10.1016/j.parkreldis.2020.07.009 32731191

[5] Grimm MJ Respondek G Stamelou M Arzberger T Ferguson L Gelpi E. Clinical conditions “suggestive of progressive supranuclear palsy” - diagnostic performance. Mov Disord. (2020) 35:2301–13. 10.1002/mds.28263 32914550 PMC7953080

[6] Kim SL Lee MJ Lee MS. Cognitive dysfunction associated with falls in progressive supranuclear palsy. Gait Posture. (2014) 40:605–9. 10.1016/j.gaitpost.2014.07.005 25088758

[7] Brown FS Rowe JB Passamonti L Rittman T. Falls in progressive supranuclear palsy. Mov Disord Clin Pract. (2019) 7:16–24. 10.1002/mdc3.12879 31970205 PMC6962663

[8] Bluett B Litvan I Cheng S Juncos J Riley DE Standaert DG. Understanding falls in progressive supranuclear palsy. Parkinsonism Relat Disord. (2017) 35:75–81. 10.1016/j.parkreldis.2016.12.009 28007518

[9] Slade SC Finkelstein D McGinley J Morris ME. Exercise and physical activity for people with Progressive Supranuclear Palsy: a systematic review. Clin Rehabil. (2020) 34:23–3. 10.1177/0269215519877235 31559853 PMC6943961

[10] Slade SC Bruce C McGinley JL Bloem BR Morris ME. Patient and care-partner views on exercise and structured physical activity for people with Progressive Supranuclear Palsy. PLoS ONE. (2020) 15:e0234265. 10.1371/journal.pone.0234265 32502214 PMC7274424

[11] Radder DLM Lígia Silva de Lima A Domingos J Keus SHJ van Nimwegen M Bloem BR. Physiotherapy in Parkinson's disease: a meta-analysis of present treatment modalities. Neurorehabil Neural Repair. (2020) 34:871–80. 10.1177/1545968320952799 32917125 PMC7564288

[12] Morris ME Menz H McGinley JL Watts JJ Huxham FE Murphy AT. A randomized controlled trial to reduce falls and improve mobility in people with Parkinson's disease. Neurorehabil Neural Repair. (2015) 29:777–85. 10.1177/1545968314565511 25567121

[13] Morris ME Taylor NF Watts JJ Evans A Horne M Kempster P. A home program of strength training, movement strategy training and education did not prevent falls in people with Parkinson's disease: a randomised trial. J Physiother. (2017) 63:94–00. 10.1016/j.jphys.2017.02.015 28342682

[14] Wittwer JE Winbolt M Morris ME. A home-based, music-cued movement program is feasible and may improve gait in Progressive Supranuclear Palsy. Front Neurol. (2019) 10:116. 10.3389/fneur.2019.00116 30837939 PMC6389624

[15] Rocha P Aguiar L McClelland JA Morris ME Dance therapy for Parkinson's disease: a randomised feasibility trial. Intern J Ther Rehabil. (2018) 25:64–2. 10.12968/ijtr.2018.25.2.64

[16] van der Kolk NM de Vries NM Kessels RPC Joosten H Zwinderman AH Post B. Effectiveness of home-based and remotely supervised aerobic exercise in Parkinson's disease: a double-blind, randomised controlled trial. Lancet Neurol. (2019) 18:998–1008. 10.1016/S1474-4422(19)30285-6 31521532

[17] Tong A Sainsbury P Craig J. Consolidated criteria for reporting qualitative research (COREQ): a 32-item checklist for interviews and focus groups. Int J Qual Health Care. (2007) 19:349–57. 10.1093/intqhc/mzm042 17872937

[18] Miles M Huberman AM Saldana J. Qualitative Data Analysis: A Methods Sourcebook. 3rd ed. Los Angeles, CA: Sage (2013).

[19] Rodriguez A Smith J. Phenomenology as a healthcare research method. Evid Based Nurs. (2018) 21:96. 10.1136/eb-2018-102990 30201830

[20] Slade SC Underwood M McGinley JL Morris ME. Exercise and Progressive Supranuclear Palsy: the need for explicit exercise reporting. BMC Neurol. (2019) 19:305. 10.1186/s12883-019-1539-4 31783740 PMC6884751

[21] Smith JA Osborn M. Interpretative phenomenological analysis as a useful methodology for research on the lived experience of pain. Br J Pain. (2015) 9:41–2. 10.1177/2049463714541642 26516556 PMC4616994

[22] Miyata H Kai I Reconsidering evaluation criteria for scientific adequacy of health care research: an integrative framework of quantitative and qualitative research. Int J Qual Methods. (2009) 8:64–75. 10.1177/160940690900800106 16813064

[23] Santiago Delefosse M Bruchez C Gavin A Stephen S Diversity of the quality criteria in qualitative research in the health sciences: lessons from a lexicometric analysis composed of 133 guidelines. Forum Qual Soc Res. (2015) 16:Art 11. 10.17169/fqs-16.2.2275

[24] Frazzitta G Maestri R Ghilardi MF Riboldazzi G Perini M Bertotti G. Intensive rehabilitation increases BDNF serum levels in parkinsonian patients: a randomized study. Neurorehabil Neural Repair. (2014) 28:163–8. 10.1177/1545968313508474 24213955

[25] Liu Y Yan T Chu JM Chen Y Dunnett S Ho YS. The beneficial effects of physical exercise in the brain and related pathophysiological mechanisms in neurodegenerative diseases. Lab Invest. (2019) 99:943–57. 10.1038/s41374-019-0232-y 30808929

[26] Johansson H Hagströmer M Grooten WJA Franzén E. Exercise-induced neuroplasticity in parkinson's disease: a metasynthesis of the literature. Neural Plast. (2020) 2020:8961493. 10.1155/2020/8961493 32256559 PMC7079218

[27] Morris ME Ellis TD Jazayeri D Heng H Thomson A Balasundaram A. Boxing for Parkinson's disease: has implementation accelerated beyond current evidence? Frontiers Neurol. (2019) 10:1222. 10.3389/fneur.2019.01222 31866923 PMC6904341

[28] Canning CG Sherrington C Lord SR Close JCT Heritier S Heller GZ. Exercise for falls prevention in Parkinson disease: a randomized controlled trial. Neurology. (2015) 84:304–12. 10.1212/WNL.0000000000001155 25552576 PMC4335992

[29] Morris ME. Movement disorders in people with Parkinson disease: a model for physical therapy. Phys Ther. (2000) 80:578–97. 10.1093/ptj/80.6.578 10842411

[30] Morris ME Iansek R Kirkwood B. A randomized controlled trial of movement strategies compared with exercise for people with Parkinson's disease. Mov Disord. (2009) 24:64–71. 10.1002/mds.22295 18942100

[31] Carroll LM Morris ME O'Connor WT Clifford AM. Is aquatic therapy optimally prescribed for Parkinson's disease? A systematic review and meta-analysis. J Park Dis. (2020) 10:59–76. 10.3233/JPD-191784 31815701

[32] Canning CG Allen NE Nackaerts E Paul SS Nieuwboer A Gilat M. Virtual reality in research and rehabilitation of gait and balance in Parkinson disease. Nat Rev Neurol. (2020) 16:409–25. 10.1038/s41582-020-0370-2 32591756

[33] Ellis T Rochester L. Mobilizing Parkinson's disease: the future of exercise. J Parkinsons Dis. (2018) 8:S95–100. 10.3233/JPD-181489 30584167 PMC6311359

[34] Rochester L Baker K Hetherington V Jones D Willems AM Kwakkel G Evidence for motor learning in Parkinson's disease: acquisition, automaticity and retention of cued gait performance after training with external rhythmical cues. Brain Res. (2010) 1319:103–11. 10.1016/j.brainres.2010.01.001 20064492

[35] Flanigan RL Professional learning networks taking off. Educ Digest Essent Read Condens Quick Rev. (2012) 77:42–45.

